# Spectroelectrochemical Enzyme Sensor System for Acetaldehyde Detection in Wine

**DOI:** 10.3390/bios12111032

**Published:** 2022-11-17

**Authors:** David Ibáñez, María Begoña González-García, David Hernández-Santos, Pablo Fanjul-Bolado

**Affiliations:** Metrohm DropSens S.L., Vivero de Ciencias de la Salud, C/Colegio Santo Domingo de Guzmán s/n, 33010 Oviedo (Asturias), Spain

**Keywords:** spectroelectrochemistry, acetaldehyde, aldehyde dehydrogenase, diaphorase, screen-printed electrodes (SPEs)

## Abstract

A new spectroelectrochemical two-enzyme sensor system has been developed for the detection of acetaldehyde in wine. A combination of spectroscopy and electrochemistry improves the analytical features of the electrochemical sensor because the optical information collected with this system is only associated with acetaldehyde and avoids the interferents also present in wines as polyphenols. Spectroelectrochemical detection is achieved by the analysis of the optical properties of the K_3_[Fe(CN)_6_]/K_4_[Fe(CN)_6_] redox couple involved in the enzymatic process: aldehyde dehydrogenase catalyzes the aldehyde oxidation using β-nicotinamide adenine dinucleotide hydrate (NAD^+^) as a cofactor and, simultaneously, diaphorase reoxidizes the NADH formed in the first enzymatic process due to the presence of K_3_[Fe(CN)_6_]. An analysis of the characteristic UV-vis bands of K_3_[Fe(CN)_6_] at 310 and 420 nm allows the detection of acetaldehyde, since absorption bands are only related to the oxidation of this substrate, and avoids the contribution of other interferents.

## 1. Introduction

Acetaldehyde (AA) is a basic compound present in certain alcoholic beverages such as wine. It is mainly produced through two processes, through the oxidation of ethanol by the action of the enzyme alcohol dehydrogenase, and also during the fermentation process by the decarboxylation of pyruvate by the enzyme pyruvate decarboxylase. Both reactions can take place throughout the wine production process, with the quantity of aldehyde produced in these processes being important. AA is currently an indicator of wine quality despite being used to control its biological aging, since it has a negative impact on the wine’s aroma when it is present in high concentrations. Furthermore, due to its toxic effects, it plays a crucial role in alcohol intoxication [1,2]. Concentration of AA in wine depends on many important factors [3,4], such as, its rapid reaction with sulfur dioxide, the grape variety, the manufacturing process, fermentation time, alcoholic strength, etc. Quantification of AA in alcoholic beverages is traditionally carried out using chemical methods based on the distillation of AA with bisulfite [5,6], colorimetry [7], chromatography [8,9] or enzymatic methods [1,10]. However, these methods show several drawbacks such as a derivatization step due to its high volatility, the necessity of weak interactions to improve the sensitivity, their susceptibility to environmental interferences, the need to decolorize, filter, or degas samples before assay, tedious and long (more than 2 h) protocols for the preparation of the sample, high costs, etc.

**Figure 1:**
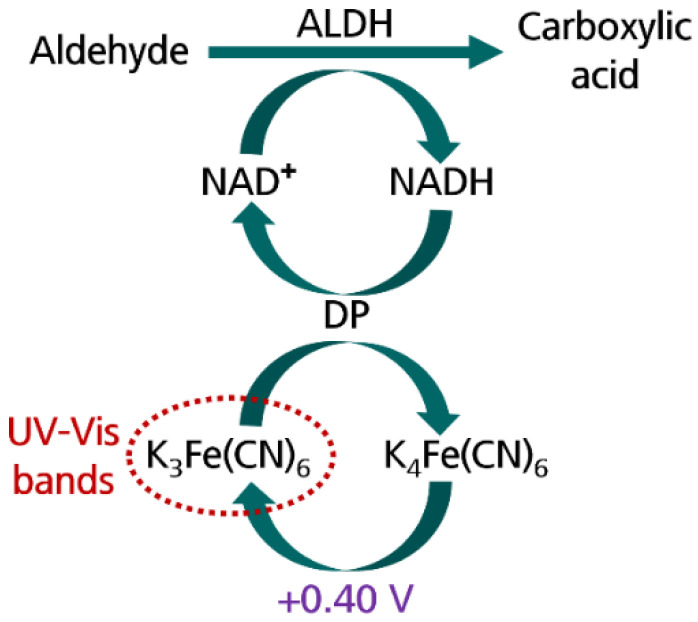
Enzymatic system formed by aldehyde dehydrogenase (ALDH), diaphorase (DP), β-nicotinamide adenine dinucleotide hydrate (NAD^+^), and K_3_[Fe(CN)_6_] which catalyzes the aldehyde oxidation to carboxylic acid.

The development of new methods for fast and easy AA detection as well as to avoid complicated instrumentation and long and complex procedures is still required. A combination of enzymatic systems with electrochemistry provides new devices for the easy and quick detection of aldehyde compounds [11,12,13]. Particularly, AA detection is based on the enzymatic system formed by the combinative work of aldehyde dehydrogenase (ALDH) and diaphorase (DP) (Figure 1). ALDH catalyzes the oxidation of aldehydes to carboxylic acids using NAD^+^ as a cofactor, and particularly, AA is oxidized to acetate. At the same time, DP reoxidizes the NADH previously generated during the ALDH reaction and reduces K_3_[Fe(CN)_6_] to K_4_[Fe(CN)_6_]. The enzymatic system is completed with the electrochemical oxidation of K_4_[Fe(CN)_6_], previously generated to K_3_[Fe(CN)_6_].

A combination of electrochemical sensors and spectroscopy has been scarcely used due to the traditional instrumental limitations of spectroelectrochemical techniques. Fortunately, the development of new setups and commercial instruments [14] enables the spectroelectrochemical detection of a variety of analytes [15,16] due to UV-vis spectroelectrochemistry and joins the advantages of electrochemistry and UV-vis spectroscopy. In a single experiment, spectroelectrochemistry provides two signals of different natures, which is a very powerful feature to obtain valuable information about the system being studied. In this enzymatic system, electrochemistry produces the oxidation of K_4_[Fe(CN)_6_], previously generated during the enzymatic process, to K_3_[Fe(CN)_6_]. The simultaneous optical monitorization takes the advantage of the optical properties of the K_4_[Fe(CN)_6_]/K_3_[Fe(CN)_6_] redox couple, allowing the quick and easy detection of AA. Furthermore, the autovalidated character of this technique provides valuable information in a single experiment [17,18]. Although the number of spectroelectrochemical sensors is increasing due to the advantages of this hybrid technique [19,20,21,22,23], enzymatic systems are rarely analyzed using spectroelectrochemical techniques.

In the present work, the usefulness of the proposed enzymatic sensor system is demonstrated for the spectroelectrochemical detection of AA. To the best of our knowledge, this is the first time that a disposable sensor combines UV-vis spectroelectrochemistry with the enzymatic system previously described, providing excellent results.

## 2. Materials and Methods

### 2.1. Reagents and Instrumentation

Aldehyde dehydrogenase, potassium activated from baker’s yeast (*S. cerevisiae*) (ALDH, EC 1.2.1.5), diaphorase from clostridium kluyveri (DP, EC 1.8.1.4), β-nicotinamide adenine dinucleotide hydrate (NAD^+^), bovine serum albumin (BSA), potassium ferricyanide (K_3_[Fe(CN)_6_]), polyvinylpolypyrrolidone (PVPP), and acetaldehyde (AA) were purchased from Merck (Sigma–Aldrich, Madrid, Spain). All chemicals were analytical grade. Aqueous solutions were prepared using ultrapure water (Direct-Q^TM^ 5 system, Millipore, Spain).

Screen-printed carbon electrodes (DRP-110, Metrohm DropSens, Oviedo, Spain) were used to perform the electrochemical experiments, while spectroelectrochemical measurements were carried out with screen-printed gold electrodes (DRP-220AT, Metrohm DropSens, Spain) in order to favor the reflection of the light on the electrode surface. The electronic systems consisted of a flat ceramic card with a circular carbon or gold working electrode (WE, 4 mm diameter), a carbon or gold counter electrode (CE), and a silver pseudoreference electrode (RE). Electrochemical measurements were performed at room temperature using a multichannel bipotentiostat, galvanostat, and impedance analyzer µStat-i Multi16 controlled using DropView 8400 M v.1.01 software in combination with an eight-channel connector and specific connectors for SPEs (DRP-4MMHCAST8 and DRP-CASTDIR respectively, Metrohm DropSens, Oviedo, Spain). Spectroelectrochemical measurements were performed with SPELEC instrument (Metrohm DropSens, Oviedo, Spain) controlled using DropView SPELEC software in combination with a bifurcated reflection probe (DRP-RPROBE-VIS-UV, Metrohm DropSens, Oviedo, Spain), and a reflection cell (DRP-REFLECELL, Metrohm DropSens, Oviedo, Spain) for working in a near-normal reflection configuration.

A fluorometric assay kit (Sigma–Aldrich, Madrid, Spain) was used to determine the concentration of acetaldehyde in white and rosé wines. Fluorometric experiments were carried out using the 532 nm laser of SPELECRAMAN532 instrument (Metrohm DropSens, Oviedo, Spain) as an excitation source, while the fluorescence signal was acquired with SPELEC.

### 2.2. Methods

#### 2.2.1. Electrochemical and Spectroelectrochemical Detection of Acetaldehyde

Electrochemical detection was performed with a drop of 60 µL on the screen-printed electrode (SPE), ensuring that the solution covers WE, RE, and CE, using chronoamperometry at +0.40 V for 60 s.

Spectroelectrochemical detection was also carried out using chronoamperometry applying +0.40 V for 300 s. In order to favor the increase of the absorption bands and their better definition, longer times were required for the spectroelectrochemical detection than for the electrochemical one. In addition, 100 µL of solution was used to ensure the spectroelectrochemical cell was filled and bubbles did not remain inside of the device. Light reflected onto the electrode surface was collected using an integration time of 1 s. UV-vis spectra were simultaneously recorded with the electrochemical measurements. According to the experimental time of the spectroelectrochemical experiments (300 s) and the integration time selected (1 s), 300 spectra were recorded during the whole measurement.

#### 2.2.2. Preparation of Wine Samples

Two Spanish wines, Cariñena white wine and Jerez rosé wine, were tested. The samples were not treated before taking the electrochemical and spectroelectrochemical measurements, and were only diluted in a 0.1 M phosphate + 0.1 M KCl buffer solution (pH 8). Calibration plots of wine dilutions were represented in terms where, for example, the value of 0.1 corresponded to the dilution of 1 mL of wine to 10 mL (1:10), 0.25 corresponded to a dilution of 2.5 mL of wine to 10 mL (2.5:10), etc. For fluorometric tests, white and rosé wine samples were diluted 100 times in ultrapure water.

#### 2.2.3. Determination of Enzyme Activities and Michaelis Constants

Enzyme activity of ALDH and DP, expressed in U (µmole of substrate transformed per minute and per mg of protein), has been calculated using spectroscopic methods [10] in previous work [24]. Briefly, ALDH activity was measured following the rate of reduction of NAD^+^ using spectroscopic monitoring for 3 min of the band at 340 nm, which was associated with NADH. DP activity was measured following the rate of reduction of K_3_[Fe(CN)_6_] by the analysis of the band at 420 nm for 3 min. Spectroscopic determination was performed in transmission configuration considering Lambert–Beer’s law (*A = ε × b × C*) and the calculated activities of ALDH and DP were 0.26 U/mg and 5.93 U/mg, respectively.

In addition, Michaelis constants for NAD^+^ and AA were also calculated in previous work by the fitting of the amperometric data to the Lineweaver-Burk model [24]. The calculated K_M_ of NAD^+^ and AA were 0.101 mM and 0.907 mM, respectively.

## 3. Results

### 3.1. Electrochemical Detection of Acetaldehyde

**Figure 2:**
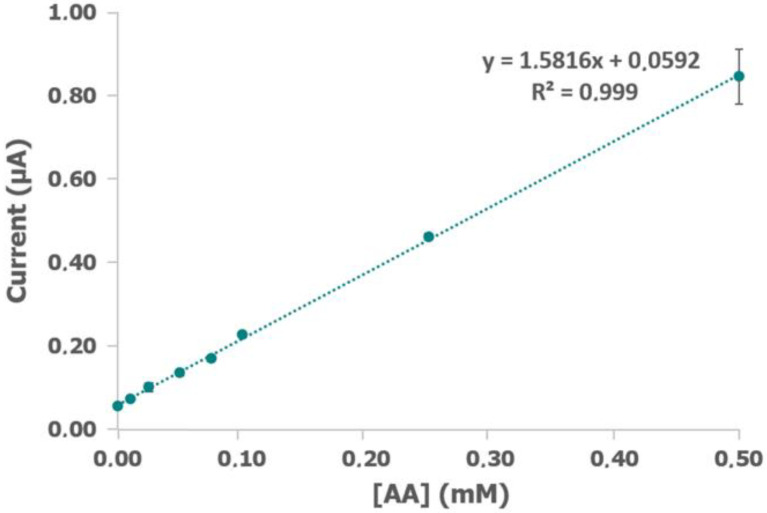
Calibration plot of current obtained after applying +0.40 V for 60 s with different concentrations of acetaldehyde (AA) and 0.07 U/mL ALDH, 0.07 U/mL DP, 1 mM NAD^+^, 1 mM K_3_[Fe(CN)_6_], and 0.1% bovine serum albumin (BSA) in 0.1 M phosphate and 0.1 M KCl buffer solution (pH 8).

Preliminary assays were considered to establish the initial experimental conditions. Electrochemical detection of AA was performed in 0.07 U/mL ALDH, 0.07 U/mL DP, 1 mM NAD^+^, 1 mM K_3_[Fe(CN)_6_], and 0.1% BSA in 0.1 M phosphate + 0.1 M KCl buffer solution (pH 8) applying +0.40 V for 60 s (electrochemical data are shown in Figure S1). Under these experimental conditions, Figure 2 shows the calibration plot obtained from 1 × 10^−5^ M to 5 × 10^−4^ M AA. As can be observed, the results fit the equation y = 1.816 + 0.0592 for this concentration range. In addition, the high correlation coefficient value (R^2^ = 0.999) and very low error bars ensures good adjustment and reproducibility.

**Figure 3:**
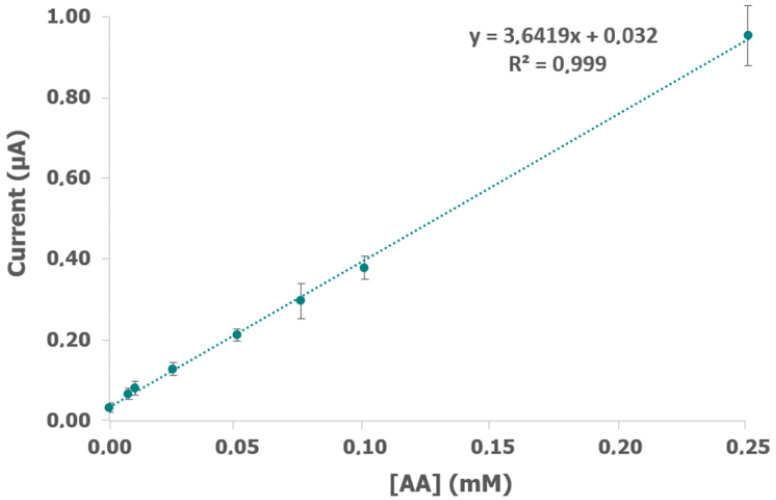
Calibration plot of AA obtained after applying +0.40 V for 60 s with different concentrations of acetaldehyde (AA) and 0.14 U/mL ALDH, 0.14 U/mL DP, 1 mM NAD^+^, 1 mM K_3_[Fe(CN)_6_], and 0.1% BSA in 0.1 M phosphate and 0.1 M KCl buffer solution (pH 8).

In order to obtain a wider concentration range, different concentrations of ALDH and DP were evaluated. The ratio between ALDH and DP is a crucial parameter because the lack of activity of one of them could hamper the reliability of the electrochemical measurements [13]. According to previous works [12,13,24], ratio ALDH/DP = 1 remains constant and both concentrations were modified in the same way. Different amounts of enzymes were evaluated, but the best results shown in Figure 3 were obtained working in in 0.14 U/mL ALDH, 0.14 U/mL DP, 1 mM NAD^+^, 1 mM K_3_[Fe(CN)_6_], and 0.1% BSA in 0.1 M phosphate + 0.1 M KCl buffer solution (pH 8) (electrochemical data are shown in Figure S2). A higher concentration of ALDH and DP did not improve the electrochemical results. The calibration curve showed a higher slope than the value obtained under the previous conditions (Figure 2), a wider concentration range (from 5 × 10^−6^ M to 2.5 × 10^−4^ M), and maintained a good adjustment (R^2^ = 0.999) and reproducibility.

Table 1 summarizes the experimental conditions and the electrochemical results in the detection of AA. According to the linear range of AA, 0.14 U/mL ALDH and 0.14 U/mL DP were selected to continue with the development of the new enzymatic device.

**Figure 4:**
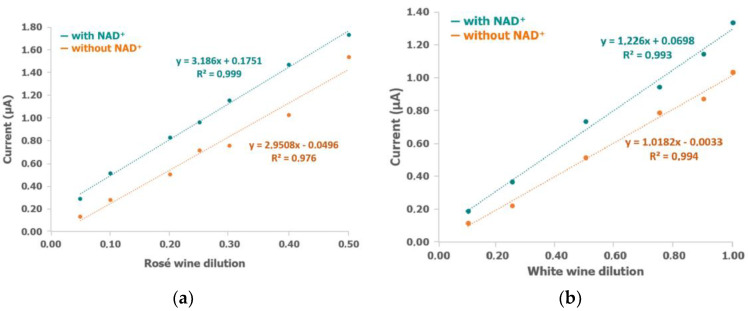
Calibration plot of (**a**) rosé and (**b**) white wines obtained after applying +0.40 V for 60 s with different dilutions of wine and 0.14 U/mL ALDH, 0.14 U/mL DP, with (green dots) and without (orange dots) 1 mM NAD^+^, 1 mM K_3_[Fe(CN)_6_], and 0.1% BSA in 0.1 M phosphate and 0.1 M KCl buffer solution (pH 8).

However, apart from AA there were more components in wine samples that could interfere in the electrochemical detection, for instance, polyphenols. Initially, calibration curves with different dilutions of rosé and white wines in buffer solution were carried out (green dots in Figure 4a,b, respectively) (electrochemical data are shown in Figure S3a,b). As can be observed, dilutions from 0.05 to 0.5 for rosé wine and 0.1 to 1 (no dilution) for white wine fit a linear curve. In addition, the same experiments were performed without a NAD^+^ cofactor in the sample (orange dots in Figure 4a,b) to remove the contribution of AA to the current. In this way, these measurements provide the electrochemical signal associated with other components but not with AA because the enzymatic reaction did not take place. Similar slopes were obtained working without NAD^+^, and the interferents contribution is clearly demonstrated since the electrochemical current was closer than the response obtained with NAD^+^. The average of the current difference observed with and without NAD^+^ was 0.29 µA and 0.19 µA for rosé and white wine, respectively.

Other parameters involved in the detection of AA were optimized to minimize the contribution of the interferents. Particularly, lower potential + 0.20 V was applied to carry out the AA calibration with and without NAD^+^. As was expected, the current value obtained was lower than when applying +0.40 V, but the contribution of the interferents in the electrochemical signal did not decrease when NAD^+^ was not added in the system (data not shown). A significant alternative to remove the phenolic compounds in wines consists of their treatment with polyvinylpolypyrrolidone (PVPP) [25,26,27]. Accordingly, 25 mL of each wine was mixed with 0.5 g of PVPP, the mixture was stirred for at least 5 min and then filtered before being used [28,29]. After this protocol, the samples were decolored due to the fact that PVPP absorbs most polyphenol present in wines. The electrochemical calibration of decolored rosé and white wines was performed under the same experimental conditions as those shown in Figure 4 (0.14 U/mL ALDH, 0.14 U/mL DP, 1 mM NAD^+^, 1 mM K_3_[Fe(CN)_6_], and 0.1% BSA in 0.1 M phosphate and 0.1 M KCl buffer solution). Although PVPP is useful to decrease the amount of phenolic compounds, the electrochemical results (data not shown) do not display good reproducibility and the accurate quantification of AA cannot be achieved. According to the results obtained, the electrochemical detection of AA based on the enzymatic ALDH/DP sensor systems seems complicated and alternative methodologies must be developed.

### 3.2. Spectroelectrochemical Detection of Acetaldehyde

Spectroscopic detection simultaneously performed with the electrochemical oxidation is an interesting methodology based on the optical properties of the enzymatic system employed, particularly those related to the K_3_[Fe(CN)_6_]/K_4_[Fe(CN)_6_] redox couple. K_3_[Fe(CN)_6_] shows two characteristic UV-vis bands at 310 and 420 nm [30], while K_4_[Fe(CN)_6_] does not show an absorption signal. The main advantage with respect to electrochemical detection is that K_3_[Fe(CN)_6_]/K_4_[Fe(CN)_6_] conversion is only related to AA and not to polyphenols or other interferents present in wine samples. In order to ensure the removal of this contribution, gallic acid was considered as representative polyphenol and its optical properties were analyzed. Gallic acid only shows one band at 280 nm, which does not interfere with K_3_[Fe(CN)_6_] bands. An additional advantage of spectroelectrochemical analysis is that the pretreatment of wine samples is not required.

**Figure 5:**
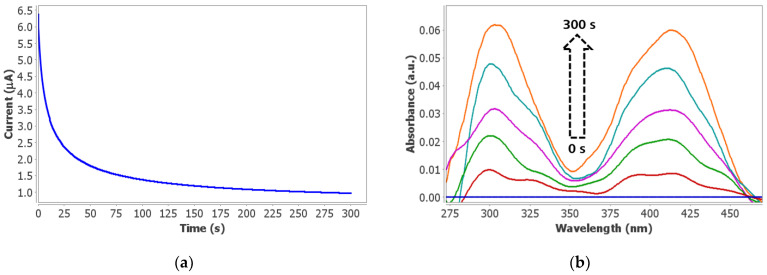
(**a**) Chronoamperometry and (**b**) UV-vis spectra recorded in 0.6 mM AA, 0.14 U/mL ALDH, 0.14 U/mL DP, 1 mM NAD^+^, 1 mM K_3_[Fe(CN)_6_], and 0.1% BSA in 0.1 M phosphate and 0.1 M KCl buffer solution (pH 8). Potential of +0.40 V was applied for 300 s.

UV-vis spectroelectrochemistry was carried out, applying +0.40 V for 300 s instead of 60 s to favor the enhancement of the optical signal. The electrochemical reaction (chronoamperogram shown in Figure 5a) produces the oxidation to K_3_[Fe(CN)_6_] while their characteristic bands are simultaneously detected (Figure 5b) during the whole measurement. Figure 5b shows the evolution of the spectroelectrochemical signal in 0.6 mM AA during 300 s. The spectrum of the initial solution, which corresponds to the K_4_[Fe(CN)_6_] generated during the enzymatic reaction, was taken as a reference (blue line in Figure 5b). As Figure 5b shows, absorbance of UV-vis bands of K_3_[Fe(CN)_6_] increases during the oxidation process of K_4_[Fe(CN)_6_]. In order to demonstrate that the observed signal corresponds to the oxidation process previously explained, the optical monitoring of the enzymatic reaction (10 min) without potential, was carried out. As can be observed in Figure S4, the absorbance decreases during the enzymatic reaction because the initial K_3_[Fe(CN)_6_] present in solution is reduced to K_4_[Fe(CN)_6_] which does not absorb in the UV-vis region.

**Figure 6:**
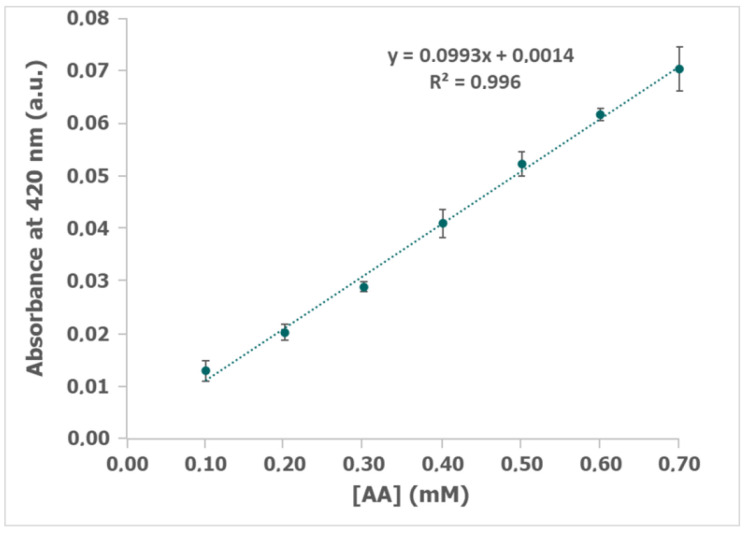
A calibration plot of absorbance at 420 nm obtained after applying +0.40 V for 300 s with different concentrations of acetaldehyde (AA) (0.1–0.7 mM) and 0.14 U/mL ALDH, 0.14 U/mL DP, 1 mM NAD^+^, 1 mM K_3_[Fe(CN)_6_], and 0.1% BSA in 0.1 M phosphate and 0.1 M KCl buffer solution (pH 8).

Different concentrations of AA were evaluated in 0.14 U/mL ALDH, 0.14 U/mL DP, 1 mM NAD^+^, 1 mM K_3_[Fe(CN)_6_], and 0.1% BSA in 0.1 M phosphate and 0.1 M KCl buffer solution. Figure S5 displays the spectrum obtained after 300 s with different concentrations of AA. The calibration curve done with the absorbance at 420 nm after 300 s (Figure 6) from 0.1 to 0.7 mM AA fits the equation y = 0.993x + 0.0014. The high correlation coefficient value (R^2^ = 0.996) ensures the good adjustment and the usefulness and sensitivity of the spectroelectrochemical method for the detection of AA in this concentration range. Furthermore, good reproducibility is demonstrated with the small error bars.

Once the AA calibration was obtained, wine samples were measured. Different dilutions of white and rosé wines were tested, allowing us to optimize the dilution factor for each type of wine. In the case of white wine, the most reproducible results which fit the calibration range were obtained with the sample 1:2 diluted, while rosé wine must be 1:4 diluted to optimize the spectroelectrochemical results. Applying these dilution factors to the wine samples, the mixture with the other reagents was achieved. After 10 min of enzymatic reaction, the spectroelectrochemical detection was performed applying +0.40 V for 300 s and UV-vis spectra were simultaneously recorded. For white wine, absorbance at 420 nm was 0.0212 +/− 0.0012 a.u. (n = 3), while for rosé wine it was 0.0287 +/− 0.0025 a.u. (n = 3). Quantification of AA was easily calculated extracting the AA concentration from the calibration curve (y = 0.993x + 0.0014, Figure 6) and considering the dilution factor for each type of wine, but also the dilution of the wine sample in the mixture with the other reagents. The concentration of AA was calculated, obtaining 1.99 mM (87.56 mg/L) in white wine and 5.51 mM (242.44 mg/L) in rosé wine. In addition, an RSD of 5.79% and 8.84% were obtained for white and rosé wine, respectively. As was previously explained, the variability in the concentration of AA present in wines is very high, since it not only depends on the type of wine (white, rosé, etc.), but also on many other factors, with certain wines able to have a concentration of up to 700 mg/L [3]. As is reported in literature, a concentration of AA in Cariñena white wine, calculated using gas chromatography–mass spectrometry, is 87.2–87.9 mg/L [31]. In terms of Jerez rosé wine, the characteristic concentration in this kind of wine is 220–380 mg/L [32]. Hence, the experimental results obtained in this work agree with the data reported in the literature using other methods, demonstrating the usefulness of the spectroelectrochemical enzymatic system.

To validate the spectroelectrochemical method, a commercial assay kit was used to determine the AA concentration in wine samples. According to the protocol, white and rosé wines were evaluated using fluorometric tests after 30 min of incubation, obtaining 2.13 ± 0.05 mM (93.72 ± 2.2 mg/L) and 5.41 ± 0.16 mM (238.04 ± 7.4 mg/L), respectively. Hence, the spectroelectrochemical results obtained with the enzyme sensor system not only agree with those reported in literature, but also with the value calculated with a commercial kit designed for that purpose (Table 2).

## 4. Conclusions

In summary, a new spectroelectrochemical enzymatic sensor has been developed in this work. A combination of electrochemistry and UV-vis spectroscopy in a single experiment allows us to improve the analytical features that both techniques have separately. The new device is based on the joint action of ALDH and DP, which produce the oxidation of AA to acetate, but also generate K_4_[Fe(CN)_6_] during the enzymatic reactions. The K_4_[Fe(CN)_6_]/K_3_[Fe(CN)_6_] redox couple has interesting optical properties, K_3_[Fe(CN)_6_] has two absorption bands, while K_4_[Fe(CN)_6_] does not have any. The spectroscopic signal obtained in the spectroelectrochemical measurements only displays the two absorption bands of K_3_[Fe(CN)_6_], and additional bands related to the interferents also present in wines are not detected. In that way, it avoids the pretreatment of the samples to remove the contribution of other compounds as polyphenols, simplifies the detection protocol, and saves time and cost. Furthermore, the easy spectroelectrochemical monitoring of the oxidation of K_4_[Fe(CN)_6_] to K_3_[Fe(CN)_6_] allows the quantification of AA. Two different wines have been evaluated in this work and, as the pretreatment was very easy, they only required an initial dilution, white wine was diluted 1:2 and rosé wine 1:4. The calibration curve with absorbance at 420 nm vs. the AA concentration, allowed the quantification of aldehyde, being 1.99 mM or 87.66 mg/L in white wine and 5.51 mM or 242.57 mg/L in rosé wine. The spectroelectrochemical results agree with the literature as well as with those obtained with a commercial kit, and in this way, this spectroelectrochemical enzymatic sensor opens new possibilities in the detection of acetaldehyde in the wine industry.

## Figures and Tables

**Table 1:** Experimental conditions for the electrochemical detection of acetaldehyde.

Electrochemical Technique	Potential	Time	ALDH	DP	NAD^+^	K_3_[Fe(CN)_6_]	BSA	AA Linear Range
Chronoamperometry	+0.40 V	60 s	0.07 U/mL	0.07 U/mL	1 mM	1 mM	0.1%	1 × 10^−5^–5 × 10^−4^ M
Chronoamperometry	+0.40 V	60 s	0.14 U/mL	0.14 U/mL	1 mM	1 mM	0.1%	5 × 10^−6^–2.5 × 10^−4^ M

**Table 2:** Comparison among several methods for the determination of acetaldehyde in Cariñena and Jerez wines.

Detection Technique	Cariñena White Wine (mg/L)	Jerez Rosé Wine (mg/L)
GC-MS	87.2–87.9 [31]	220–380 [32]
Fluorometry	93.72 (this work)	238.04 (this work)
Spectroelectrochemistry	87.56 (this work)	242.44 (this work)

## Supplementary Materials

The following are available online at https://www.mdpi.com/article/10.3390/bios12111032/s1, Figures S1 and S2: Chronoamperograms obtained applying +0.40 V for 60 s with different concentrations of AA, ALDH, DP, NAD^+^, K_3_[Fe(CN)_6_] and BSA in phosphate and KCl buffer solution (pH 8). Figure S3: Chronoamperograms obtained applying +0.40 V for 60 s with different dilutions of rosé and white wines, ALDH, DP, NAD^+^, K_3_[Fe(CN)_6_] and BSA in phosphate and KCl buffer solution (pH 8). Figure S4: UV-vis spectra recorded during the enzymatic reaction (10 min) in AA, ALDH, DP, NAD^+^, K_3_[Fe(CN)_6_] and BSA in phosphate and KCl buffer solution (pH 8). Figure S5: UV-vis spectra obtained after applying +0.40 V for 300 s with different concentrations of AA, ALDH, DP, NAD^+^, K_3_[Fe(CN)_6_] and BSA in phosphate and KCl buffer solution (pH 8).
