# Supplementary material for: Spectroelectrochemical Enzyme Sensor System for Acetaldehyde Detection in Wine
Source: Biosensors (Basel). 2022 Nov 17;12(11):1032. doi: 10.3390/bios12111032 (PMC9688840; doi:10.3390/bios12111032)
Supplement: Supplementary file 1 [file biosensors-12-01032-s001.zip › biosensors-2004883-supplementary.pdf]

biosensors

MDPI

Article

Spectroelectrochemical enzyme sensor system for acetaldehyde detection in wine

David Ibáñez *, María Begoña González-García, David Hernández-Santos, Pablo Fanjul-Bolado *

Metrohm DropSens S.L., Vivero de Ciencias de la Salud, C/Colegio Santo Domingo de Guzmán s/n, 33010, Oviedo (Asturias), Spain

* Correspondence: david.ibanez@metrohm.com (D.I.); pablo.fanjul@metrohm.com (P.F.-B.)

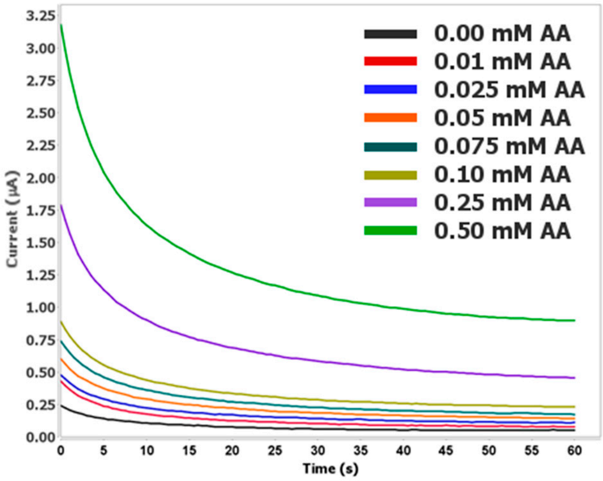

Figure S1. Chronoamperograms obtained applying + 0.40 V for 60 s with different concentrations of AA, 0.07 U/mL ALDH, 0.07 U/mL DP, 1 mM NAD^+, 1 mM $K_3[Fe(CN)_6]$, and 0.1 % BSA in 0.1 M phosphate and 0.1 M KCl buffer solution (pH 8).

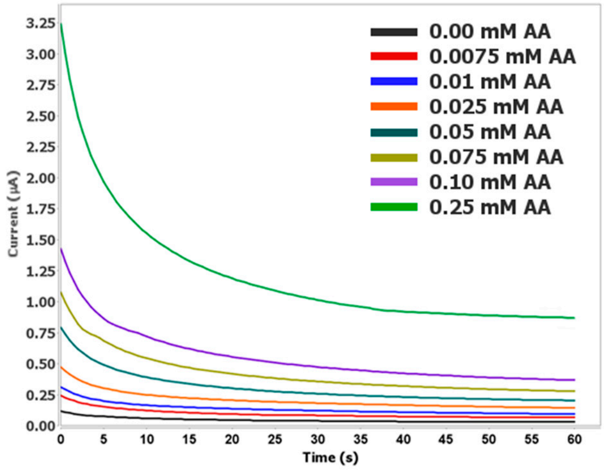

Figure S2. Chronoamperograms obtained applying + 0.40 V for 60 s with different concentrations of AA, 0.14 U/mL ALDH, 0.14 U/mL DP, 1 mM NAD^+, 1 mM $K_3[Fe(CN)_6]$, and 0.1 % BSA in 0.1 M phosphate and 0.1 M KCl buffer solution (pH 8).

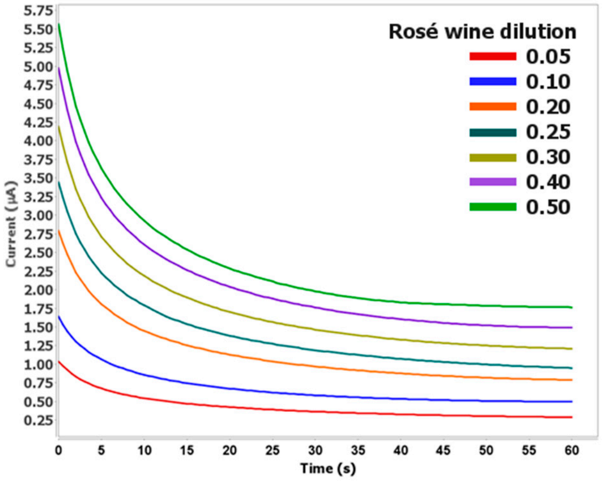

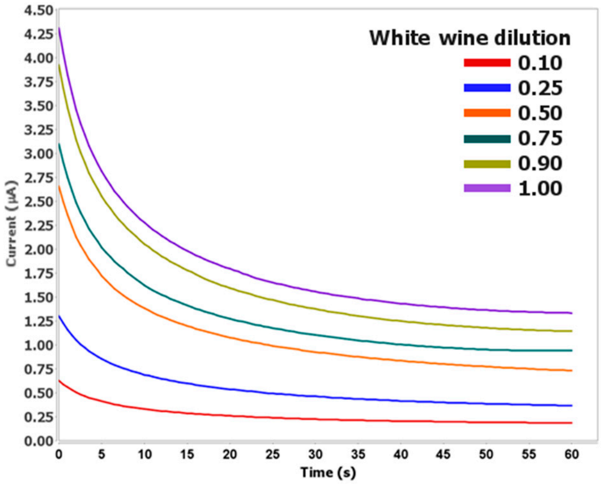

Copyright: © 2022 by the authors. Licensee MDPI, Basel, Switzerland. This article is an open access article distributed under the terms and conditions of the Creative Commons Attribution (CC BY) license (https://creativecommons.org/licenses/by/4.0/).

(a) **(b)**

Figure S3. Chronoamperograms obtained applying + 0.40 V for 60 s with different dilutions of (**a**) rosé, and (**b**) white wines, 0.14 U/mL ALDH, 0.14 U/mL DP, 1 mM NAD^+, 1 mM $K_3[Fe(CN)_6]$, and 0.1 % BSA in 0.1 M phosphate and 0.1 M KCl buffer solution (pH 8).

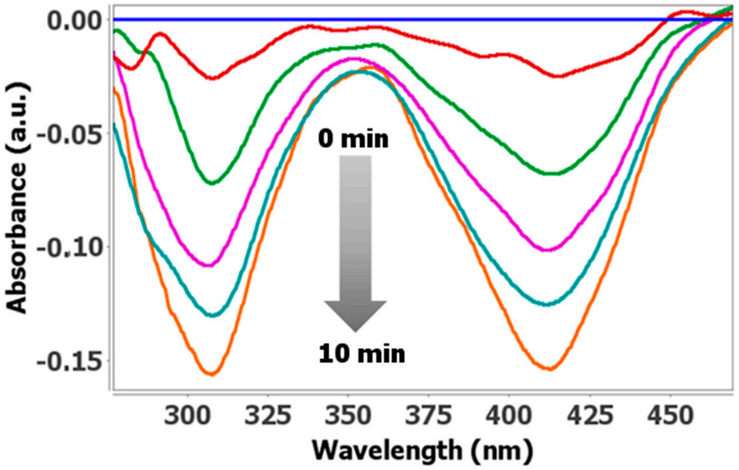

Figure S4. UV-vis spectra recorded during the enzymatic reaction (10 min) in 0.6 mM AA, 0.14 U/mL ALDH, 0.14 U/mL DP, 1 mM NAD^+, 1 mM $K_3[Fe(CN)_6]$, and 0.1 % BSA in 0.1 M phosphate and 0.1 M KCl buffer solution (pH 8). Potential was not applied in this experiment.

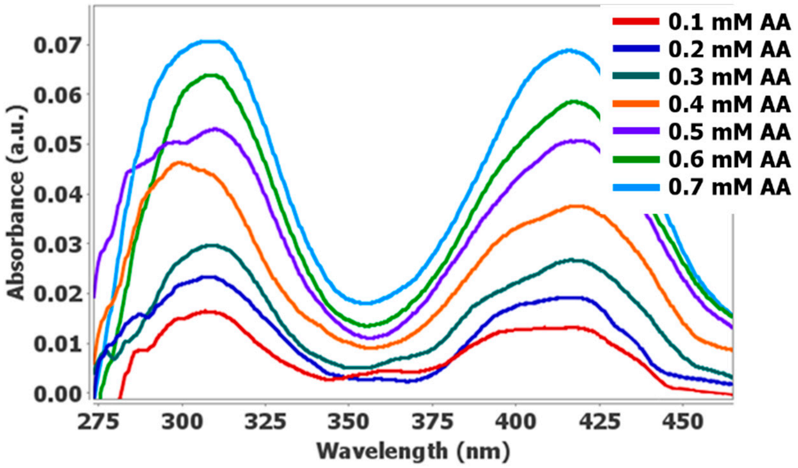

Figure S5. UV-vis spectra obtained after applying + 0.40 V for 300 s with different concentrations of AA (0.1-0.7 mM) and 0.14 U/mL ALDH, 0.14 U/mL DP, 1 mM NAD^+, 1 mM $K_3[Fe(CN)_6]$, and 0.1 % BSA in 0.1 M phosphate and 0.1 M KCl buffer solution (pH 8).
